# Coding systems and monitoring practices across the ERN ReCONNET: insights from a comprehensive survey and unmet needs

**DOI:** 10.1186/s13023-026-04304-7

**Published:** 2026-03-30

**Authors:** Matilde Bandeira, Diana Marinello, Sofia C. Barreira, Jutta G. Richter, J. K. de Vries-Bouwstra, Ingrid E. Lundberg, Cristina Pamfil, Alain Cornet, Marta Mosca, Ana Rath, Massimo Triggiani, Gabriele Simonini, Alessandro Ferraris, Wiktoria Solecka, Marco Lanzillotta, Francesca Regola, Jiří Vencovský, Vincent Sobanski, Camelia Bucșa, Michel Tsang-A-Sjoe, Jakub Závada, Nicoletta Del Papa, Zana Brkic, Nicolas Hunzelmann, Isabell Haase, Charissa Frank, Vanessa Smith, Rosaria Talarico, João Eurico Fonseca, Matthias Schneider

**Affiliations:** 1 Rheumatology Department, Unidade Local de Saúde Santa Maria, Centro Académico Médico de Lisboa (CAML), Lisbon, Portugal; 2 Gulbenkian Institute for Molecular Medicine, CAML, Lisbon, Portugal; 3 Rheumatology Unit, Department of Clinical and Experimental Medicine, Azienda Ospedaliero Universitaria Pisana, University of Pisa, Pisa, Italy; 4 Division of Rheumatology and Systemic Inflammatory Diseases, Department of Medicine, University Medical Center Hamburg-Eppendorf, Hamburg, Germany; 5 Hiller Research Unit Rheumatology, Medical Faculty, Heinrich-Heine- University, Universitätsklinikum Düsseldorf, Düsseldorf, Germany; 6 Department of Rheumatology, Leiden University Medical Centre, Leiden, Netherlands; 7 Department of Gastroenterology, Dermatology and Rheumatology, Theme Inflammation and Aging, Division of Rheumatology, Department of Medicine, Karolinska University Hospital, Karolinska Institutet, Solna, Stockholm, Sweden; 8 Department of Rheumatology, Emergency County Clinical Hospital Cluj, Iuliu Hațieganu University of Medicine and Pharmacy, Cluj-Napoca, Romania; 9 Lupus Europe, Brussels, Belgium; 10 Inserm, US14-Orphanet, Paris, France; 11 Division of Allergy and Clinical Immunology, AOU San Giovanni di Dio e Ruggi d’Aragona, University of Salerno, Salerno, Italy; 12 Rheumatology Unit, ERN-ReCONNET Centre, Meyer Children’s Hospital IRCCS, Florence, Italy; 13 Laboratory of Medical Genetics, Department of Experimental Medicine, Sapienza University and San Camillo Forlanini Hospital, Rome, Italy; 14 Department of Connective Tissue Diseases, National Institute of Geriatrics, Rheumatology and Rehabilitation, Warsaw, Poland; 15 Università Vita-Salute San Raffaele, Milan, Italy; 16 Unit of Immunology, Rheumatology, Allergy and Rare Diseases (UnIRAR), IRCCS San Raffaele Scientific Institute, Milan, Italy; 17 Rheumatology and Clinical Immunology Unit - ERN ReCONNET Centre, ASST SpedaliCivili, Brescia, Italy; 18 Institute of Rheumatology, Prague, Czech Republic; 19 Service de Médecine Interne, U1286 - INFINITE-Institute for Translational Research in Inflammation CHU Lille, University Lille, Inserm, Lille, France; 20 Romanian Association of Relapsing Polychondritis Patients, Cluj-Napoca, Romania; 21 Faculty of Pharmacy, Iuliu Hatieganu University of Medicine and Pharmacy, Amsterdam UMC, Amsterdam, The Netherlands; 22 Department of Rheumatology, Amsterdam Rheumatology and Immunology Center, Amsterdam UMC, Amsterdam, The Netherlands; 23 Scleroderma Clinic, UOC Clinica Reumatologica, ASST Pini-CTO, Milano, Italy; 24 Department of Internal Medicine, Division of Allergology and Clinical Immunology, Erasmus Medical Centre, Rotterdam, The Netherlands; 25 Department of Dermatology and Venereology, University of Cologne, Cologne, Germany; 26 Flemish Association for Hereditary Connective Tissue Disorders, Koersel, Belgium; 27 Department of Internal Medicine, Ghent University, Ghent, Belgium; 28 Department of Rheumatology, Ghent University Hospital, Ghent, Belgium; 29 Unit for Molecular Immunology and Inflammation, VIB Inflammation Research Centre (IRC), Ghent, Belgium; 30 Rheumatology Unit, Azienda Ospedaliero Universitaria Pisana, Via Roma 67, Pisa, 56126 Italy

**Keywords:** European Reference Network, ERN ReCONNET, Coding systems, Registries, rCTDs, Rare diseases

## Abstract

**Background:**

Rare and complex connective tissue diseases (rCTDs) pose significant challenges for healthcare systems due to the lack of standardized approaches for recording and monitoring patient data. The European Reference Network on Rare and Complex Connective Tissue and Musculoskeletal Diseases (ERN ReCONNET) aims to harmonize patient data recording practices across member centres. This study evaluates current practices and unmet needs related to coding systems and monitoring.

**Methods:**

A cross-sectional study design was employed, involving three phases. The first phase, mapped the unmet needs for coding systems and monitoring practices across ERN ReCONNET centres. The second phaseprioritized these unmet needs, and the third phase aimed at defining action points to address those unmet needs by means of a Level of Agreement. We conducted a web‑based survey among ERN ReCONNET centres to evaluate current practices(response rate 75.0% [48/64] in Phase 1); and an additional inquiry in Phase 2 (58 responders) to identify the unmet needs of the Network. Data were analysed using descriptive statistics.

**Results:**

The initial survey included responses from 48 centres. About 42% of the centres used some form of paper-based records, and only 25% used dedicated biobank software. ICD-10 was predominantly applied for coding diagnoses, sub-diagnoses, and comorbidities, while ORPHAcodes were also employed, though less frequently. Clinicians were primarily responsible for data collection and submission, leading to significant administrative burden. The second survey, with 58 respondents, identified the lack of adoption of ORPHAcodesand the heterogeneity in coding systems as top unmet needs. Additionally, the survey highlighted the significant burden on clinicians and the need for improved information technology(IT) infrastructure to facilitate data extraction for ERN yearly monitoring. Lastly, 7 action points were identified in order to plan tangible actions to address the unmet needs of the rCTDs community.

**Conclusion:**

This study highlights the heterogeneity in data documentation practices, coding system usage, and monitoring activities across ERN ReCONNET centres. The findings underscore the need for standardization and optimization of data management processes. Addressing these challenges through digitalization and standardized coding practices is essential for realizing a truly integrated and collaborative network for rare connective tissue diseases across Europe.

**Supplementary Information:**

The online version contains supplementary material available at 10.1186/s13023-026-04304-7.

## Background

Rare connective tissue and musculoskeletal diseases pose a significant challenge for the healthcare systems of the European Union (EU) as they strive to deliver consistent and high-quality care. For this reason, the European Commission (EC) launched the European Reference Networks (ERNs): virtual networks involving healthcare providers (HCPs) across Europe that aim to facilitate discussion on complex and/or rare diseases and conditions that require highly specialised treatment.The European Reference Network on Rare and Complex Connective Tissue and Musculoskeletal Diseases (ERN ReCONNET) [1] stands as one of the 24 ERNs established by the EC [2], which are periodically monitored through a set of key performance indicators known as the ERN Continuous Monitoring and Quality Improvement System.

The mission of ERN ReCONNET is to develop a multi-stakeholder framework for the delivery of high quality, innovative, sustainable and equitable standard of care and practice for better access to care of European patients with rare connective tissue diseases(rCTDs) [1, 3].Considering the need to promote cross-border data collection and analyses that can lead to more robust research and ultimately can strongly support the improvement of care, the EC has launched the development of 24 registries related to the disease areas of each ERN. For this reason, ERN ReCONNET created TogethERN ReCONNET [4], the European Registry Infrastructure for data harmonization in rCTDs. The aims of TogethERN ReCONNET include (i) promoting a harmonised data collection approach on rCTDs in Europe, (ii) integrating and implementing existing rCTDs data, (iii) improving disease knowledge, clinical management and care provided to rCTDs patients, (iv) facilitating rCTDs research, post-authorisation studies and cost-effective healthcare planning.

Among thedifferent initiatives of the Network, the ERN ReCONNET Working Group on Registries and eHealth has promoted the mapping of the current coding systems adopted by the ERN ReCONNET centres, both Full Members (FMs) or Affiliated Partners (APs), for different domains of care.

The harmonization of coding systems within ERN ReCONNET centres is, in fact, a pivotal step towards enhancing the quality and consistency of healthcare data, ultimately improving patient care, and informing health policy for rCDTs [5].

The peculiarities of rare and complex diseases require documentation and monitoring practices to be harmonized and standardized in these domains to ensure high-quality, consistent care across different countries and healthcare systems. This harmonization is particularly challenging given the diverse healthcare policies, resources, and infrastructures across Europe. Thus, based onthese considerations, ERN ReCONNET contributed to this important unmet need by providing an overview of the current coding systems and ERN monitoring practices adopted in the ERN ReCONNET centres, besides outlining potentialaction pointsdeveloped by the Network and that are reported in this paper.

## Methods

This studywas conducted with a multi-steps approach that has foreseen:

1. A first explorative phase, consisting in a cross-sectional analysis of the existing coding systems and ERN monitoring practices adopted in ERN ReCONNET centres;
2. A second phase which focused on the identification and prioritisation of the unmet needs of the whole ERN ReCONNET (HCPs and patient representatives involved in the Network) regarding the rCTDs coding and the ERN Monitoring systems;
3. A third phase that defined an action plan based on the evidence emerged in steps 1 and 2.
4. During thefirst phase, a web-based survey was developed to collect data on the coding systems and monitoring practices employed within ERN ReCONNET centres. The survey was designed collaboratively within the framework of the ERN ReCONNET‘Registry and eHealth’ working group, with input from healthcare professionals, data specialists, and patient representatives to ensure relevance and comprehensiveness.

The survey was distributed electronically to the ERN ReCONNET healthcare providersusing EUSurvey [6], an open‑source platform developed and maintained by the European Commission. Respondents were provided with a link to access the survey, ensuring confidentiality and traceability of responses. One invitation per ERN ReCONNET centre was sent to the PI or their designated data manager (*n* = 64).

The first survey, developed in English, consisted of 51 questions structured in sections focusing on the following domains: i) the coding systems (Supplementary Table 2) utilized within ERN ReCONNET centres in different settings (outpatientand inpatient) regarding diagnosis, sub-diagnosis, comorbidities and procedures; ii)the type of documentation of clinical, laboratory and imaging data; iii)the ERN monitoring practices employed for rCTDs, with a focus onthe accessibility of data from the hospital’s information system, and the coding systems currently used for the collection of data related to the 18 Key Performance Indicators(KPIs) of the annual ERN monitoring activities. The survey was launchedin May 2023 and was available for three months.

In the second phase, the objective wasto prioritize the unmet needsidentifiedin the first phase.A short online survey was anonymouslydistributedto the whole ERN ReCONNET (HCPs and patient representatives involved in the Network, within the European Patient Advocacy Groups [ePAGs])allowing responders to rank the uncovered unmet needs in order of relevance, by means of a drag-and-drop ranking procedure.

Additionally, challenges related to ERN Monitoring data collection were identified and ranked.The second survey was opened in May 2024 for 2 weeks.

Quantitative data from the surveys’ responses (Phase 1 and 2) were analysed to derive descriptive statistics using Microsoft Excel.

In the third phase, the results of phases 1 and 2 were discussed in 3 online meetings of the ERN ReCONNET Registries and eHealth Working Group conducted from December 2023 and June 2024, in order to identify the preliminary action points that were finally agreed upon by means of a level of agreement (LoA); briefly, the edited draft of the action points was distributed to the Registries and eHealth Working Group via EU survey, asking to indicate their agreement with each statement with “yes” or “no” and to vote on their level of agreement (LoA), using a scale of 0–10 (0 indicating no agreement at all and 10 indicating full agreement). A consensus was accepted if > 75% (threshold pre-agreed) of the members voted in favour of each statement. The mean and the standard deviation of the LoA, as well as the percentage of the Working Group members with an agreement ≥ 8, were reported.

It is important to highlight that, following the collaborative and patient partnership nature of ERN ReCONNET, the involvement of ePAGs was consistent and substantive throughout all phases of the work. Specific patient representatives participated as members of the ERN ReCONNET Registry Working Group, namely representatives of relapsing polychondritis, Ehlers-Danlos syndrome, and systemic lupus erythematosus. These have contributed to the design of the surveys, helped interpret the findings, the unmet needs identification and raking, as well as reviewing the manuscript draft. Furthermore, all patient representatives included in ERN ReCONNET were invited to answer the second survey, in order to obtain more widespread feedback, across different countries and diseases. To this end, the embedded collaboration with patient representatives ensured that their perspective shaped the strategic framing of the work.

## Results

### Phase 1

The survey was distributed to the 64 Healthcare Providers that were involved in ERN ReCONNET at the time of the launch of the survey (55 FMs and 9 APs) and a total of 48 replies were collected (Supplementary Table 1); the response rate was 75%. In total, 92% of the replies were from ERN ReCONNET FMs and 8% from ERN ReCONNET APs.The 48 responding centres represented 18 European countries, notably including Italy (*n* = 8), Germany (*n* = 6), and the Netherlands (*n* = 5, Supplementary Table 1).

Regarding domain i) particularlypatient data documentation during daily clinical practice, the majority (58%) utilized electronic health records. Notably, just 1 centre(2%) adhered to complete paper-based documentation, while 40% employed hybrid methods, with 42% using some form of paper-based patient files.

When asked about clinical information systems (CIS), with the possibility of multiple simultaneous choices, most respondents (88%, *n* = 42) reported using a standard CIS of the hospital (HIS). Tencentres (21%) described an adapted standard CIS for rheumatology or a rheumatology-specific CIS (Table 2). Fifteencentres used both a standard CIS and a Rheumatology-adapted or -specific CIS. Seven centres (15%) selected the “other” option. From these, five centres mentioned specific CIS for genetics (such as DNALAB or SPICE [7]) or for other areas such as surgical or intensive care. Some centres mentioned specific CIS such as national databases for rare diseases, Excel^®^files, and electronic patient dossiers for specific diseases such as systemic sclerosis or Ehlers-Danlos syndrome.The CIS referredby the respondents were almost all different.

**Table 1:** Clinical Information Systems (CIS) used in different ERN ReCONNET centres

Clinical information system	Link/reference
BAMARA/BNDMR	https://www.fimatho.fr/en/research-and-financing/bndmr
CGM Medico	https://www.cgm.com/irl_en/products/hospital-clinic.html
DxCare	https://www.omnia-health.com/product/healthcare-4
Electronic patient dossier (EPD)	https://www.ictrecht.nl/en/legal-advice/electronic-health-records-epd-ecd
EOS, provided by DNALAB	http://www.dnalab.it
EPIC	https://www.epic.com/software/
Glintt: Desktop medico/EPR	https://globalcare.glintt.com/clinical/
HiX: clinical care	https://www.chipsoft.com/nl-NL/oplossingen/505
iSoft	https://i-soft.us/medical.html
Klinisch Werkstation (KWS)	https://www.nexuzhealth.com/nl/oplossingen/kws-ziekenhuizen
Metavision PDMS	https://www.imd-soft.com/metavision-products/mv-for-intensive-care-adults-neonatal-and-pediatric
Orbis healthcare system	https://www.dedalus.com/anz/our-offer/products/orbis-the-foundation-for-modern-digital-healthcare/
Reuma.pt database (Rheumatology-specific CIS)	https://reuma.pt
Rheumatology BCB register (Rheumatology-specific CIS)	https://bcbmedical.com/disease-specific-platform/
SAP	https://www.sap.com/
Sclinico	https://www.spms.min-saude.pt/2020/07/sclinico-hospitalar/
SPICE (genetic-specific CIS)	https://www.niaid.nih.gov/research/spice
SILLAGE	https://www.sib.fr/services-metiers/medical/sillage/

Only 21% (*n* = 10) reported holding data in a cloud-based system. On the contrary, 56% of respondents (*n* = 27), referred that the data collected during clinical practice was not stored in a cloud-based system. The remainder (23%, *n* = 11) did not know how data was deposited in their centre.

Moreover, the laboratory information management system software was also heterogenous (Table 1). LOINC [8] was only used in 19 centres (40%,Supplementary Table 2).

**Table 2:** Laboratory information systems used by the different ERN ReCONNET centres

Laboratory information system	*n*
Hospital-based platform	7
Medat	6
GLIMS provider MIPS	3
Same platform as CIS	2
DNLab	2
AlchymiA	1
Rocket^®^ CorVu	1
HUS Lab	1
Laboweb	1
Infinity	1
Entrepot de données de santé	1
Maxdata	1
Insiel Data Lab	1
Galileo e-health solutions	1
ClinidataXXI	1
Modulab	1
OSM	1
Labka	1
Imed	1
LabTrain	1
Orbis	1
MediLab	1
eDeia Lab from Segilac	1
eLAB	1
OpenLIMS	1
PATHOX (GENYX version, specific for medical genetics laboratories)	1
Molis by CGM	1
EPIC	1
Areas Engineering	1
Unknown	4

Regarding the storing of images, the use of common picture archiving and communication system (PACS)resources between centres was slightly higher as compared to clinical and laboratory information, with 8 centres using *Sectra* (17%). Other frequently used systems were *Philips* and *Centricity PACS* (13%, *n* = 6 each), *AGFA* and *Siemens* (8%, *n* = 4 each). *Fujifilm* was reported in 2 centres (4%) and *Infinitt* in 1 (2%). Nevertheless, 19 centres chose the option “other”.

In terms of capturing and/or managing biological samples, only 25% (*n* = 12) of the respondents reported using dedicated biobank software. Almost half of the respondents (48%, *n* = 23) reported not using this kind of software, and the remainder did not know and did not provide an answer.

**Fig. 1:**
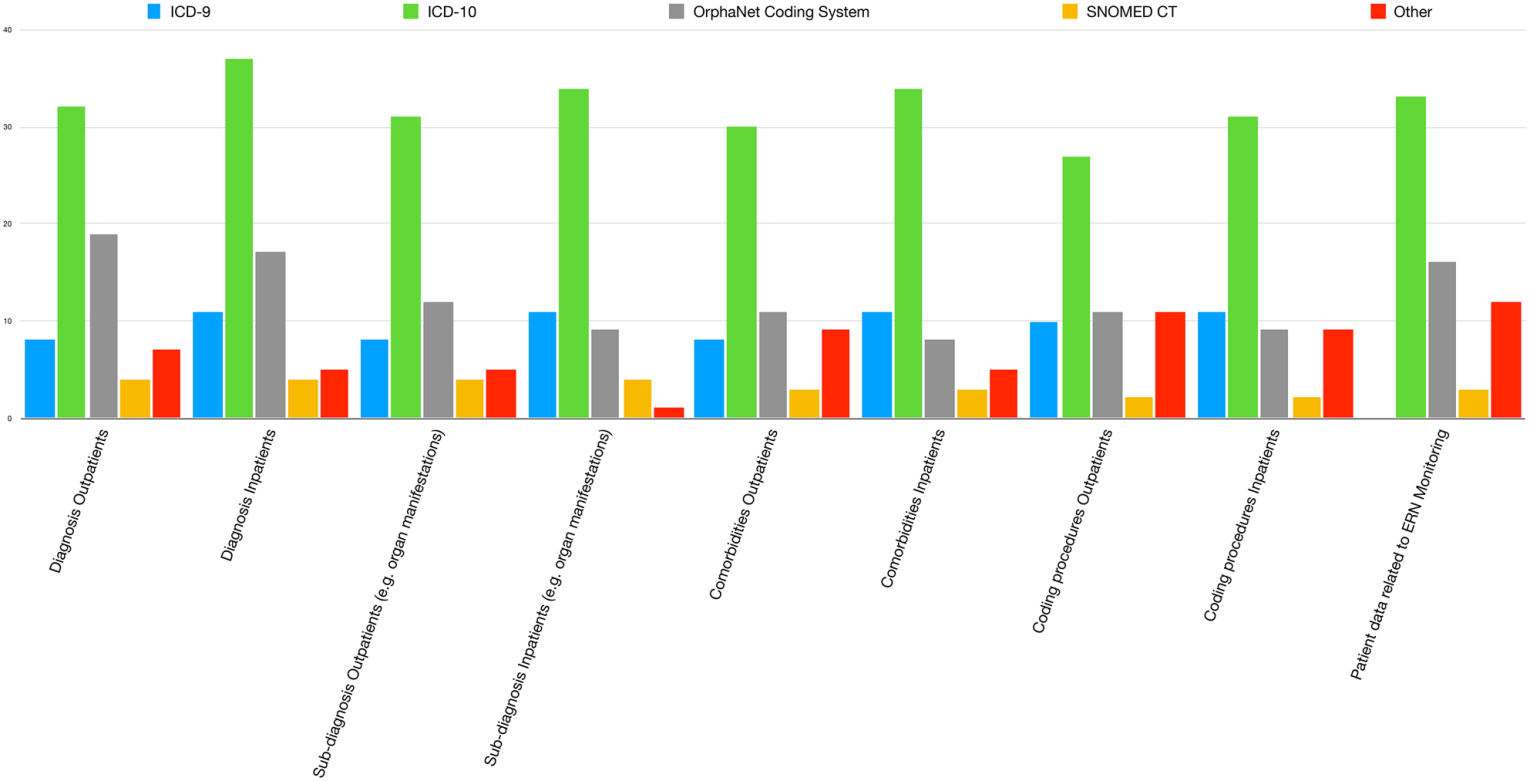
Distribution of coding systems used in each centre for different contexts

Regarding the coding systems used for different contexts (diagnosis, sub-diagnosis such as specific organ manifestations, comorbidities and procedures, both for inpatients and outpatients), ICD was the most frequently used coding system. ICD-10 was by far the most frequently applied coding system, with some centres still using ICD-9. ORPHAcodeswere used less often (Fig. 1). The centres that reported the use of other systems describedtheir own patient’s database/clinical chart, based on diagnosis and not on a code, others reportednationally-defined codes for diagnosis or their national Rheumatology dedicated registries in order to confirm diagnoses (such as Reuma.pt [9] and the Italian National Rare Diseases Registry [10]).

**Fig. 2:**
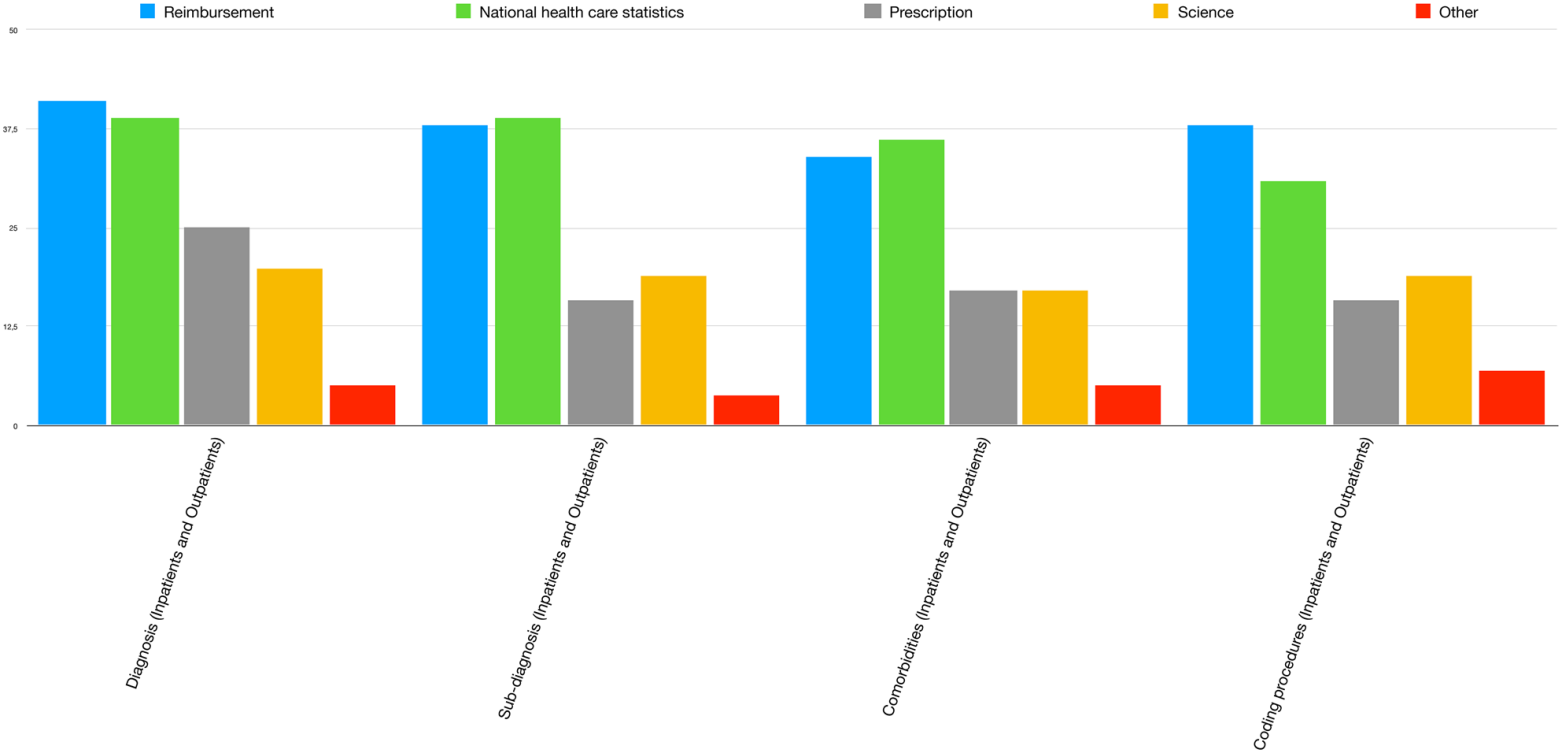
Purposes for coding in different contexts

Many different purposes were stated for the use of coding systems (Fig. 2); the most frequent ones across all situations were reimbursement and national health care statistics, with prescription and research being less frequent reasons for using coding systems.

**Fig. 3:**
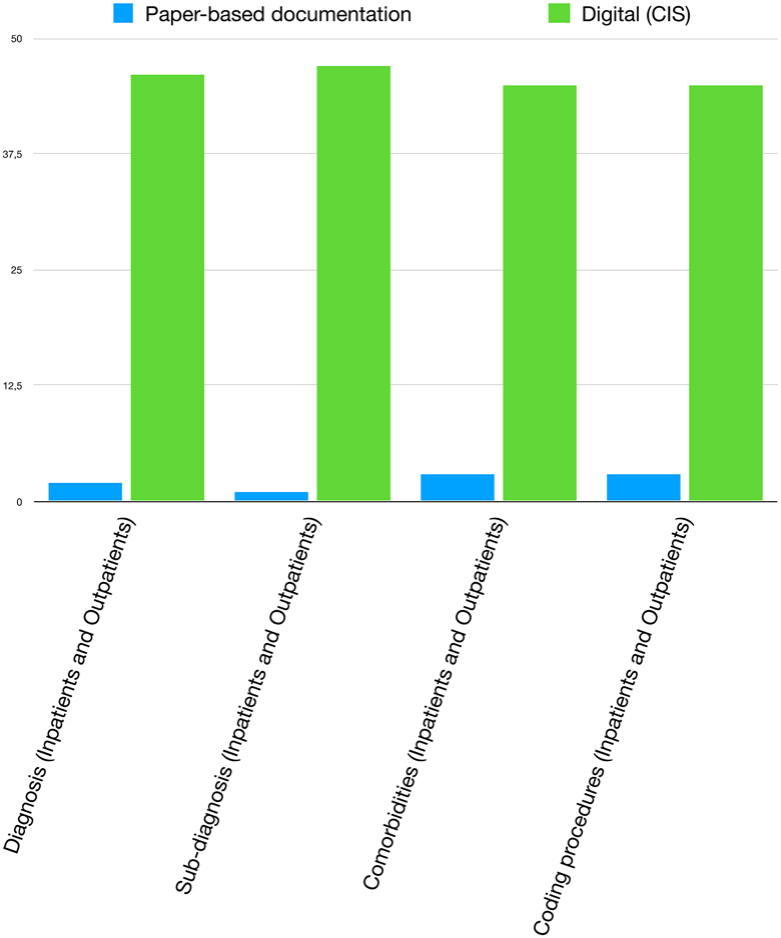
Methods of documentation of coding for different contexts

Notably, in every context surveyed, at least one centre reported using structured digital documentation for patient codification (Fig. 3).

When asked whether the patient data was digitally transferred into registries, half of the respondents denied having this assistance (50%, *n* = 24). From the24 centres that had data digitally transferred into registries, 11 reported that this transfer of data was voluntary or mandatory (for instance, for national death registries), in the laterwith an automatic processin five centres (21%, 10% of all centres).

Regarding domain ii), the coding systems adopted for monitoring activities in ERN ReCONNET (Fig. 1) were similar to the ones used in each centre for clinical management, with ICD-10 being the most widely used, followed by ORPHAcodes. Most centres reported being able to take some information directly from their CIS to fill out the annual report for ERN ReCONNET. However, only 23 (48%) could export complete data on the number of rCTDs patients and only 16 (33%) exportedcomplete data on the number of procedures performed in their centre. Frequently, the information extracted was only partial (40%, *n* = 19, and 52%, *n* = 25, for the number or patients and procedures, respectively). A minority of centres wasnot able to access these numbers directly from the CIS (13%, *n* = 6, and 15%, *n* = 7, respectively).

The vast majority stated that a clinician or healthcare professional from their unit was responsible for both the collection (60%, *n* = 29) of this data and submission for the ERN monitoring system (85%, *n* = 41). In the other centres, either this was performed by a non-clinician unit staff member (collection 23%, *n* = 11, and submission 10%, *n* = 5) or, less frequently, a staff member from the hospital administration (collection 17%, *n* = 8, and submission 4%, *n* = 2).

### Phase 2

For the second phase, 58 respondents participated, consisting primarily of healthcare professionals working in ERN ReCONNET centres (95.1%) and a small number ofpatient representatives (4.9%). The results of the second survey revealed critical insights into the prioritization of unmet needs regarding coding systems and ERN monitoring practices within ERN ReCONNET, that are summarised in Tables 3 and 4, respectively. In particular, Table 3 presents how six main unmet needs related to coding systems were ranked by centres. Respondents assigned a position from 1st (highest priority) to 6th (lowest). To facilitate interpretation, the most frequently selected top-three priorities are highlighted in bold. Although some statements may conceptually overlap, they were presented separately as they emerged distinctly from the previous survey phase. Table 4 presents how six key unmet needs related to ERN ReCONNET monitoring activities were prioritized by the respondents. Each item was ranked from 1st (highest priority) to 6th (lowest), and the table shows the percentage of respondents assigning each position to each item. For easier interpretation, the three most frequently selected top priorities in each row are highlighted in bold. Some of the listed challenges may overlap in scope (e.g., lack of IT systems and lack of dedicated staff), but are presented separately here as they were identified as distinct themes during the first survey phase.

**Table 3:**
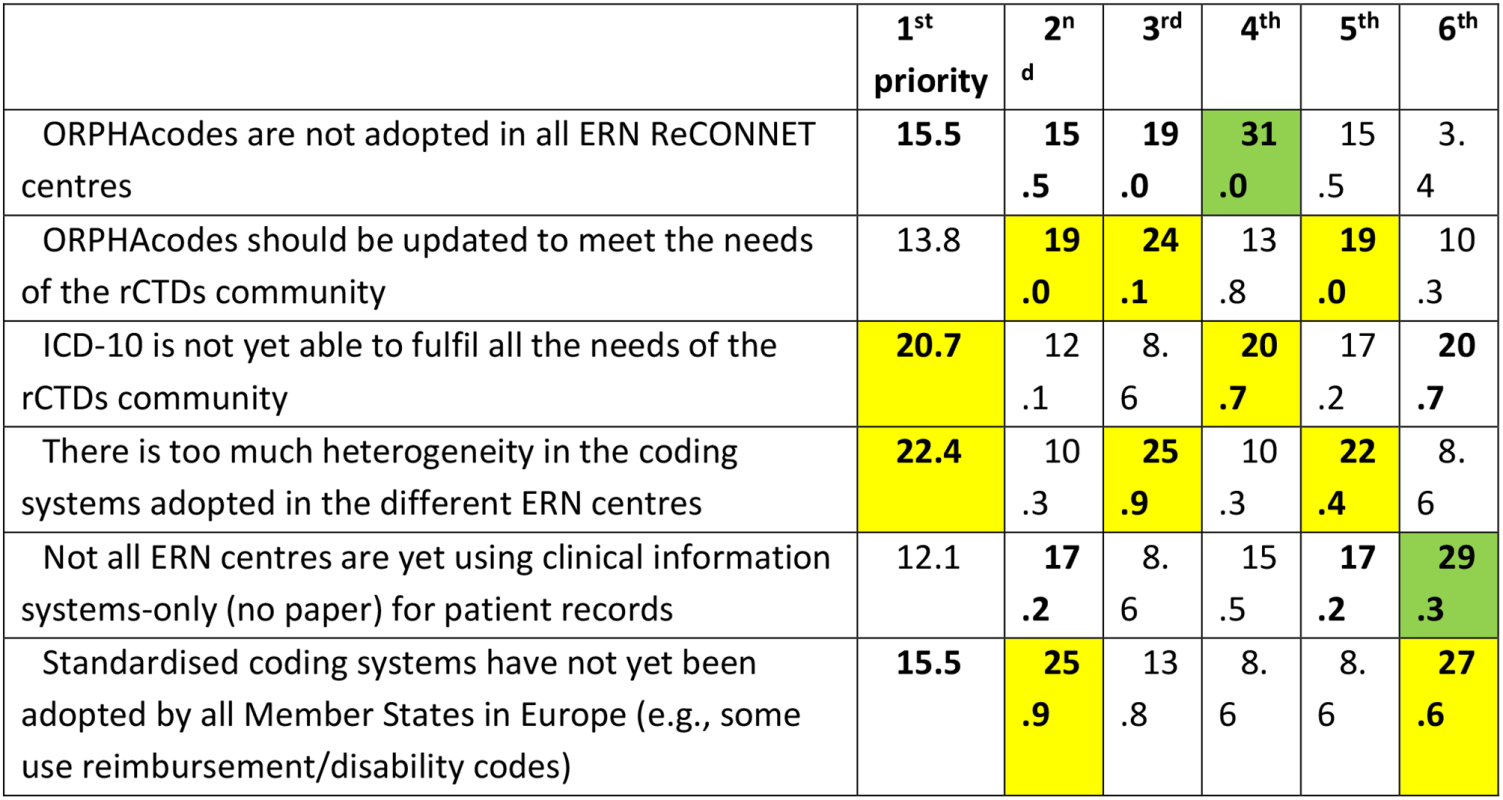
Unmet needs on ERN ReCONNET coding systems and how the second survey respondents prioritized them (in %); Yellow: no dominated suggestion, green: dominated suggestions

**Table 4:**
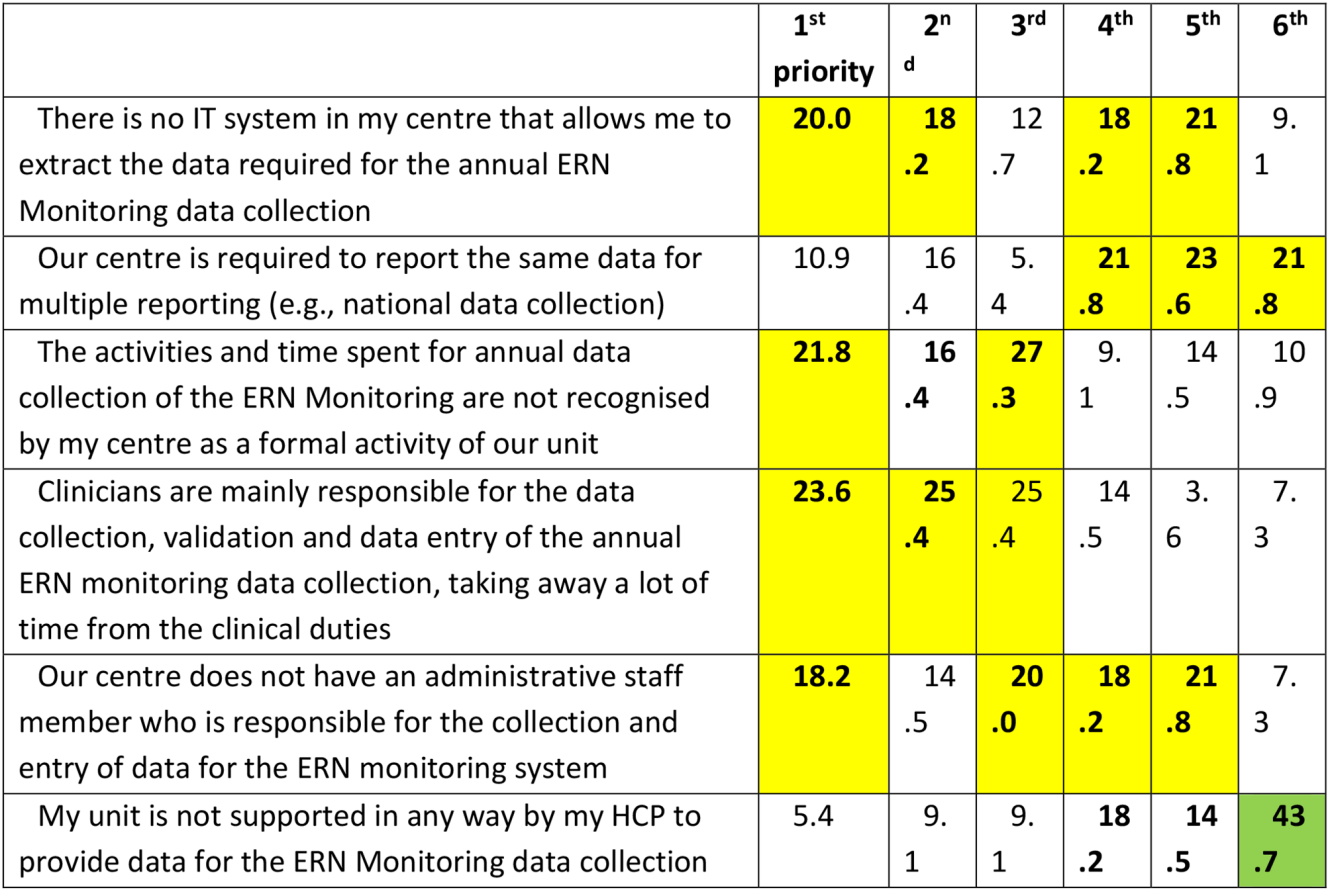
Unmet needs on ERN ReCONNET monitoring activities and how the second survey respondents prioritized them (in %)

### Phase 3

The discussion on the results of the previous phases allowed us to identify 7 action points that were finally agreed by means of a LoA (Table 5).Each preliminary action point was explicitly linked to the barriers identified in Phase 2, responsible actors were defined (e.g., ERN ReCONNET Member States, OrphaNet) and an implementation timeline suggested (short6–12 months, medium: 12–36 months, long: >36 months).

**Table 5:** ERN ReCONNET Action Points on coding systems in rCTDs and on ERN monitoring practices

	Action Points	LoA mean (SD)	≥8/10 (%)	Barrier addressed	Responsible actor	Suggested timeline
1	There is an urgent need to update of ORPHAcodes to adapt them to the needs of the rCTDs community.	8 (2)	68	Gaps in available ORPHAcodes for rCTDs and comorbid conditions	OrphaNet and ERN ReCONNET	Short-Medium
2	The adoption of standardised coding systems (e.g., ORPHAcodes, disability codings) can be promoted in all centres treating rCTDs across all European Member States, starting with the ERN HCPs.	9 (1)	79	Heterogeneity in coding systems among centres	ERN ReCONNET for the promotion of coding systems adoption; Member States for their implementation	Long
3	An assessment of the future ICD-11 coding is encouraged to ensure the fulfilment of rCTDs community needs.	8 (2)	74	ICD‑10 lacks granularity for rare diseases	WHO ICD-11 Taskforce, collaboration from ERN ReCONNET and other ERNs	Medium
4	The promotion of the use of electronic clinical information systems should be encouraged, progressively reducing paper-based patient records.	10 (1)	89	Persistent use of paper‑based records	ERN ReCONNET	Medium
5	Member States should promote the allocation of appropriate resources to all ERN HCPs in order to enable their participation in the ERN activities (e.g., registry data enter, monitoring, etc.).	9 (1)	89	Lack of allocated resources for ERN activities	ERN ReCONNET Member States	Long
6	Member States are encouraged to promote the creation of ERN Offices at all ERN HCPs in order to support the respective centres to participate in all ERN-related activities.	9 (1)	84	Fragmented coordination and high clinician administrative burden	ERN ReCONNET Member States	Medium - Long
7	ERN HCPs need to have available appropriate IT systems and coding practices to facilitate the reporting for the ERN Monitoring system.	9 (1)	95	IT limitations that impede automated data reporting	Hospital management of ERN HCPs	Medium-Long

LoA: Level of Agreement. SD: standard deviation

The current version of the ORPHAcodesavailable for rCTDs comprises different levels of disease coding, however, ERN ReCONNET experts express the need to update the coding currently available in order to enable the promotion of their adoption across Europe *(89% agreed, first round*, *n* = 19).

Thanks to the European Reference Networks and to their integration in the different European Member States, standardised coding systems can be promoted in the different centres treating rCTDs. The adoption can support the increase of standardisation and interoperability of data across Europe, which can lead to better research and management of rCTDs *(100% agreed*,* first round*, *n* = 19).

Currently, the ICD-11 coding system is being developed. After its release, it will be important to assess the new system to understand whether it addresses the needs of the rCTDs community or if any further refinement is required *(95% agreed*,* first round*, *n* = 19).

In order to promote an increase in the interoperability of data, HCPs treating rCTDs should progressively reduce the usage of paper-based patient records and increase the use of electronic clinical information systems *(100% agreed*,* first round*, *n* = 19).

As far as the ERN monitoring practices are concerned and more broadly on the ERN system, it is crucial to ensure that ERN HCPs are allocated with appropriate resources that allow them to participate in the ERN activities (with focus on ERN registry and ERN monitoring data collection)*(95% agreed*,* first round*, *n* = 19).

Besides allocating the appropriate resources to the individual ERN clinical units, creating dedicated offices at the different ERN HCPs would support their participation in all the ERN-related activities, ensuring better coordination at HCP level and more sustainability in the long term (*100% agreed*,* first round*, *n* = 19).

In the framework of the annual ERN monitoring data collections, the ERN HCPs encounter different barriers. The availability of appropriate ITand coding systems in all the ERN HCPs could strongly support the provision of data of each HCP in a timely and less time-consuming manner *(100% agreed*,* first round*, *n* = 19).

## Discussion

In this work, we have identified the main unmet needs regarding the coding systems and ERN monitoring practices in the ERN ReCONNET and defined a set of proposed action points to be developed in the future to address the unmet needs identified.

Harmonizing patient data recording and coding systems across the EU is a strategic priority to advance research, enhance patient care, and inform health policy for rCTDs and implementing the ERNs. This includes the need of a better harmonisation of coding systems within ERN centres, also considering the crucial role that this synchronisation can have in advancing research, improving patient care, and informing health policy for rCTDs.

In the evolving landscape of healthcare, the significance of efficient and accurate coding systems cannot be overstated, particularly in the realm of rCTDs. These systems are not only pivotal for the standardization of diagnoses and treatments but also play a crucial role in facilitating Findable, Accessible, Interoperable, and Reusable (FAIR)research [11], healthcare planning, and appropriate resource allocation.

In our work, it was found that around 41% of the ERN ReCONNET centres still use, at least partially, some variation of paper-based clinical records. This significantly challenges the way data is stored and shared between centres. It may also increase the likelihood of data and information loss.

Similarly, the fact that only 25% use a dedicated biobank software to capture and/or manage biological samples highlights both a need and an opportunity to ensure the possibility of connecting/exchanging this data, that would allow for an optimization of collaborative research within ERN ReCONNET centres.

The basis for all interactions, comparisons, and collaboration between centres is a standardized coding of disease entities.The International Classification of Diseases (ICD) [5], the most widely used system with international standardization, offers a comprehensive classification of diseases and health-related conditions. As expected, most centres use ICD-10. For rCTDs, this means improved comparability of data, and better epidemiological tracking. However, the ICD system’s broad categories are sometimes lacking the granularity needed for rare diseases, potentially leading to underrepresentation or inaccurate coding of these conditions.

In addition, procedures that are part of the reporting in ERNs are not captured by ICD-10. This is reported to have been improved in the upcoming ICD-11 [5].

The Systematized Nomenclature of Medicine – Clinical Terms (SNOMED CT) [12] renowned for its detailed clinical vocabulary which provides an extensive range of terms for diseases, clinical findings, and procedures, is rarely implemented due to complexity and vastness. Specifically designed for rare diseases, ORPHAcodes [13] present a unique system that complements the ICD and SNOMED CT. They offer a high level of detail and specificity for rare conditions, including rCTDs. However, one major drawback is that ORPHAcodesare not yet universally adopted, potentially leading to inconsistencies in coding and data collection across different regions and healthcare systems [14]. Some centres useORPHAcodes [13] as recommended by the EU for rare diseases. However, since someORPHAcodeslack diagnoses for rather common comorbidities that frequently affect rCTDs patients and are important in their management, a collaboration with Orphanet and ERN ReCONNET is in place to address these aspects and improve the available ORPHAcodesfor rCTDs. This is particularly important for creating the TogethERN ReCONNET, especially considering the obligation of all ERN Registries to adopt ORPHAcodes. Furthermore, cross‑mapping between ICD‑10, ORPHAcodes, and SNOMED CT is partially supported through OrphaNet’s online crosswalk tool; however, granularity mismatches—particularly for comorbidities and procedural codes—mean some concepts cannot be fully translated without loss of specificity. Of relevance for future developments in this field it is important to highlight that the coding system used in a specific centre is usually, at least partially, decided by the hospital administration or even national committees, being thus an administrative pregiven prerequisite.

One frequent concept of collaboration, especially in rare diseases, are patient registries, of which more than 100 are active in this field, across Europe [15].However, our survey demonstrates that registries often duplicate work for physicians who want to participate in multicentric research, who frequently experience a very high administrative burden related to the need to enter data from the same patient(s) in multiple registries. The ability to cross-reference clinical data with registries or local databases, as reported by some respondents, may significantly facilitate the TogethERN ReCONNET, the ERN ReCONNET monitoring but also patient data sharing for FAIR research. It is, however, currently insufficiently perceived by the low number of centres that report a non-manual transfer of data from their CIS into a registry. Additionally, the high proportion of centres where healthcare professionals, whether clinicians or not, are responsible for both data collection (60%) and submission (85%) for ERN monitoring procedures underscores the considerable time invested by these professionals outside of direct expert patient care. This demands a need to minimize such administrative burdens to optimize healthcare delivery, which can be addressed in different ways. Establishing a local ERN office at each centre was proposed to centralize data collection, streamline sharing across units, and reduce clinicians’ administrative burden.

Another point discussed for the improvement of the data collection is also the adoption of ORPHAcodesin the CIS of all the ERN centres, which should simplify the data collection for both the ERN Monitoring system and for the data entry in the TogethERN ReCONNET. Lastly, the formal recognition of the time spent by the clinicians involved in the ERNs within their HCPs could strongly support an improvement in the care provided, as it would lead to better resource allocation during the planning of the healthcare delivery in each unit, allowing clinicians to contribute to the ERN activities without having the current impact on the care provision as well as on their personal time, which is very often used to participate in the ERN activities. The process of the integration of the ERNs into national healthcare systems, should address the need to organise central support in each ERN HCP, the recognition of the ERN clinician’s time in contributing with their time and resources to the ERN mission and the harmonization of the systems used in the ERN HCPs, which should ultimately contribute to the main goal of the Joint Action.

The results of the second phase further emphasized the need for standardization and harmonization of coding systems within ERN centres. The most relevantunmet need identified was the lack of adoption of ORPHAcodesacross all ERN ReCONNET centres, with over 30% of respondents ranking it as their top or second priority. Additionally, 22.4% of respondents highlighted the heterogeneity in coding systems as a critical issue, indicating a strong need for a standardized approach.

The survey also revealed significant concerns regarding the burden of data collection and monitoring on clinicians, with 23.6% identifying this as the primary issueand almost half ranking it among their top three concerns. These findings underscore the pressing requirement for improved infrastructure, administrative support, and streamlined processes to enhance data collection efficiency and reduce the administrative burden on clinical staff.

Coding and monitoring practices are not solely technical issues but are deeply rooted in structural contexts. National health policies may mandate different primary coding systems (e.g., those designed for reimbursement vs. clinical care), while hospital administrations maydetermine resource allocation for IT infrastructure, training, and data entry. In addition, funding disparities across Member States or even regions affect a centre’s ability to implement and maintain digital systems.These underlying structures shape how and whether harmonization can occur. Therefore, efforts to standardize coding systems and streamline monitoring must be coordinated with health system leaders, policymakers, and funding bodies to ensure sustainable and equitable integration across the ERN.

Table 5 is designed as a practical tool to guide multiple stakeholders, including ERN coordinators, national health authorities, and healthcare providers, in prioritising actions for improved codification and monitoring. The proposed steps align with broader European initiatives such as the European Health Data Space (EHDS) and the European Rare Disease Registry Infrastructure (ERDRI), supporting convergence between clinical networks and policy frameworks.

While standardized coding is vital, there is a risk that overly rigid systems may ‘invisibilize’ patients whose clinical journeys do not neatly fit predefined categories. Conversely, the absence of codification may lead to administrative invisibility, limiting access to services, inclusion in research, and long-term care planning. Thus, while standardisation must be implemented carefully to avoid oversimplification, its absence can equally result in exclusion from essential systems of recognition and support.Individualization remains key in patient care at each centre, however global standardization will help build better standard care for rare diseases.

Considering the different challenges and unmet needs identified, the 7 action points proposed by ERN ReCONNET can be considered a crucial starting point for both Member States and the rCTDs community in order to address those unmet needs with practical actions. However, through physicians’ efforts, administrative changes are necessary at the country level to address all the 24 ERNs and the corresponding rare diseases and patients suffering from them.To this end, ERN ReCONNET is already planning a series of initiatives to promote the implementation of the 7 action points in collaboration with different initiatives and competent authorities (e.g.,Orphanet, Member States, etc.) with the main aim of improving the care and management of rCTDs, and in parallel, promote the integration of ERNs at national levels, which is considered a key topic for the rare and complex conditions at the European level. ERNs should not wait for the universal solution, and are an important source for multicentric solutions. One look into the future may be artificial intelligence (AI), that may be used to extract relevant information from diverse digital patient records [16] and correlate different coding systems. FAIR data are not the result of, but the basis for AI to optimize care of patients with rare and complex diseases.

While this analysis is grounded in the specific context of ERN ReCONNET and connective tissue diseases, several identified principles – such as the need for shared codification standards, transparent registry governance, and structured patient involvement – are applicable across other ERNs. Nonetheless, recommendations related to disease-specific data elements and registry interoperability may require adaptation to each network’s clinical and organisational specificities.

To translate these action points into practice, ERN HCPs and Member Centres should establish dedicated training programs for clinical coders, allocate ring‑fenced funding for IT infrastructure upgrades, and formalize institutional support that recognize ERN activities as part of official clinical roles.

## Conclusion

Harmonizing patient data records is imperative for advancing FAIR research, clinical care, and health policies, particularly in the context of rare and complex conditions like rCTDs. This study highlights the existing barriers and unfinished tasks necessary to progress in patient data sharing within ERN ReCONNET. The second survey underscored the critical importance of adopting standardized coding systems, such as ORPHAcodes, across all ERN centres and addressing the significant administrative burdens faced by clinicians involved in data collection and monitoring.

Digitalizing all patient records represents a crucial initial step towards optimizing the functioning of the ERN ReCONNET. However, significant challenges remain, particularly in integrating distinct data sources with individual codes and inputs. Standardizing coding systems and promoting interoperability also across IT systems are therefore fundamental to harmonize patient data records and facilitate seamless data sharing between all ERN ReCONNET centres.The forthcoming implementation of the European Health Data Space is expected to further streamline data interoperability across ERN HCPs. By mandating standardized data formats and secure cross‑border data exchange, the EHDS aligns closely with TogethERN ReCONNET objectives, enabling more efficient FAIR research and reducing administrative burden.A further step will be the finalisation of the TogethERN ReCONNET Registry Platform across all EU centres, as an important milestone that will promote harmonisation, but will also require rigorous efforts to overcome technical and organizational hurdles.

In conclusion, this study underscores the critical importance of harmonizing patient data records within ERN ReCONNET to drive advancements in rare and low-prevalence disease management and improve patients’ outcomes. Addressing these challenges and embracing digitalization and standardized coding practices will be pivotal in realizing the vision of a truly integrated and collaborative network for rCTDs across Europe. ERN ReCONNET will contribute to this goal with dedicated actions, while joining forces with other relevant stakeholders in order to address the unmet needs identified.

## Electronic Supplementary Material

Below is the link to the electronic supplementary material.

Supplementary Material 1

## Data Availability

The results of the survey analysed during the current study are available from the corresponding author on reasonable request.

## Declarations

### **Disclaimer**

The content of this publication represents the views of the authors only and it is their sole responsibility; it cannot be considered to reflect the views of the European Commission and/or the Health and Digital Executive Agency (HaDEA) or any other body of the European Union. The European Commission and the Agency do not accept any responsibility for use that may be made of the information it contains.
