# Supplementary material for: Coding systems and monitoring practices across the ERN ReCONNET: insights from a comprehensive survey and unmet needs
Source: Orphanet J Rare Dis. 2026 Mar 30;21:186. doi: 10.1186/s13023-026-04304-7 (PMC13154656; doi:10.1186/s13023-026-04304-7)
Supplement: Supplementary file 1 — Supplementary Material 1 [file 13023_2026_4304_MOESM1_ESM.docx]

*Supplementary Table 1–List of centres that answered the survey*

| Centre | Country | Centre type |
| --- | --- | --- |
| A.S.L. Torino – Hub S. Giovanni Bosco | Italy | Full Member |
| AO Padua | Italy | Full Member |
| AO San Camillo Forlanini – Rome | Italy | Full Member |
| AOU di Modena | Italy | Full Member |
| AOU Meyer di Firenze | Italy | Full Member |
| AOU Pisana | Italy | Full Member |
| AOU S. Giovanni di Dio e Ruggi D’Aragona | Italy | Full Member |
| APHP, HôpitalBicêtre | France | Full Member |
| APHP, HôpitalPitié-Salpêtrière | France | Full Member |
| APHP, Hôpital Raymond Poincaré | France | Full Member |
| ASST Centro Specialistico Ortopedico Traumatologico Gaetano Pini | Italy | Full Member |
| Azienda Ospedaliera Universitaria Integrata di Verona | Italy | Full Member |
| Azienda Ospedaliero Universitaria delle Marche | Italy | Full Member |
| Azienda Sanitaria Universitaria Friuli Centrale (ex ASUIUD ora ASUFC) | Italy | Full Member |
| Centro Hospitalar de Lisboa Central, EPE | Portugal | Full Member |
| Centro Hospitalar de Lisboa Norte, EPE | Portugal | Full Member |
| Centro Hospitalar do Porto, EPE | Portugal | Full Member |
| CharitéUniversitätsmedizin Berlin | Germany | Full Member |
| Children’s Clinical University Hospital, Riga | Latvia | Affiliated Partner |
| CHU de Lille | France | Full Member |
| CHU de Bordeaux | France | Full Member |
| Civil Hospital – Brescia | Italy | Full Member |
| County Emergency Clinical Hospital Cluj | Romania | Full Member |
| Erasmus MC: University Medical Centre Rotterdam | Netherlands | Full Member |
| Fondazione IRCCS Casa Sollievodella Sofferenza | Italy | Full Member |
| Fondazione Policlinico Tor Vergata Roma | Italy | Full Member |
| General Hospital of Athens Laiko | Greece | Full Member |
| Helsinki University Hospital, Hospital District of Helsinki and Uusimaa | Finland | Full Member |
| HôpitauxUniversitaires de Strasbourg | France | Full Member |
| Hospital Clínic de Barcelona y Hospital de Sant Joan de Déu | Spain | Full Member |
| Hospital of Lithuanian University of Health Science KaunoKlinikos | Lithuania | Affiliated Partner |
| Hospital UniversitariValld’Hebron | Spain | Full Member |
| Hospital Universitario 12 de Octubre | Spain | Full Member |
| Institute of Rheumatology, Prague | Czech Republic | Full Member |
| IRCCS AOU San Martino – Genoa | Italy | Full Member |
| Karolinska Universitetssjukhuset | Sweden | Full Member |
| KerckhoffKlinik | Germany | Full Member |
| Leiden University Medical Center | Netherlands | Full Member |
| Mater Dei Hospital | Malta | Affiliated Partner |
| National Institute of Geriatrics, Rheumatology and Rehabilitation (NIGRiR) | Poland | Full Member |
| Pauls Stradins Clinical University Hospital, Riga | Latvia | Affiliated Partner |
| Radboud University Medical Centre Nijmegen | Netherlands | Full Member |
| Rigshospitalet | Denmark | Full Member |
| Universitätsklinikum Düsseldorf | Germany | Full Member |
| University Hospital Ghent | Belgium | Full Member |
| University Hospitals Saint-Luc | Belgium | Full Member |
| University Medical Center Amsterdam | Netherlands | Full Member |
| UZ Leuven | Belgium | Full Member |

Supplementary Table 2 - Overview of Key Coding Standards in ERN ReCONNET: Purpose, Contents, and Application

|  | Full name | Purpose | Contains | Application in ERN ReCONNET |
| --- | --- | --- | --- | --- |
| ICD-10/11 | International Classification of Diseases | Global diagnostic classification of diseases & causes | Alphanumeric codes for diagnoses & procedures | Primary system for coding diagnoses, comorbidities, procedures |
| ORPHAcodes | Orphanet Rare Disease Codes | Detailed rare‑disease–specific classification | Unique codes for > 9000 rare diseases | Used to code rCTDs with finer granularity than ICD |
| LOINC | Logical Observation Identifiers Names and Codes | Standardize laboratory and clinical observations | Codes for lab tests, clinical measurements | Adopted by 40% of centres for laboratory data interoperability |
| SNOMED CT | Systematized Nomenclature of Medicine – Clinical Terms | Comprehensive clinical terminology | > 350000 clinical concepts (findings, procedures, body structures) | Limited implementation in ERN HCPs due to complexity; potential for future use |
